# Enhancing Meat Quality and Nutritional Value in Monogastric Livestock Using Sustainable Novel Feed Ingredients

**DOI:** 10.3390/foods14020146

**Published:** 2025-01-07

**Authors:** José A. M. Prates

**Affiliations:** 1 CIISA-Centro de Investigação Interdisciplinar em Sanidade Animal, Faculdade de Medicina Veterinária, Universidade de Lisboa, Av. da Universidade Técnica, 1300-477 Lisboa, Portugal; japrates@fmv.ulisboa.pt; 2 Associate Laboratory for Animal and Veterinary Sciences (AL4AnimalS), Av. da Universidade Técnica, 1300-477 Lisboa, Portugal

**Keywords:** monogastric animals, meat quality, novel feed ingredients, alternative protein sources, insect meal, microalgae, seaweeds, biofortified grains, agro-industrial residues, sustainable livestock production

## Abstract

This study explores the potential of novel feed ingredients for monogastric animals, such as pigs and poultry, to enhance meat quality and nutritional value while reducing the environmental footprint of production. Innovative feed options like black soldier fly larvae, *Schizochytrium* microalga, *Laminaria* seaweed, fermented soybean hulls, fortified flaxseed and grape pomace have significantly improved meat quality and nutritional traits. Results indicate that these ingredients enrich meat with omega-3 fatty acids, antioxidants, vitamins and minerals, enhancing nutritional value while improving sensory traits such as flavour, tenderness and colour. For instance, including *Laminaria* seaweed increased iodine content by up to 45%, while *Schizochytrium* microalga improved omega-3 deposition by over 70%. The inclusion of grape pomace enhanced oxidative stability and extended meat shelf life. This review also discusses the influence of ingredient composition, inclusion levels and processing techniques, alongside challenges such as regulatory constraints, ingredient cost and palatability. The alignment of these alternative feeds with circular economy principles and sustainability goals further emphasizes their role in reducing environmental impact. By summarising recent advancements, this paper underscores the transformative potential of novel feed ingredients in advancing monogastric meat production towards greater nutritional quality, sustainability and consumer acceptance.

## 1. Introduction

The global demand for high-quality animal protein has placed monogastric livestock production, particularly pigs and poultry, at the forefront of food security efforts [1]. These livestock species are recognized for their efficiency in converting feed into meat, making them crucial for addressing protein needs in developed and developing nations [2]. However, the sustainability of conventional monogastric production systems is increasingly under scrutiny due to environmental concerns, resource competition and health implications. Traditional feeds, predominantly based on maize and soybean meal, rely on finite agricultural resources and have a high environmental footprint, including deforestation, biodiversity loss and greenhouse gas emissions [3]. Additionally, the import dependence of soybean meal in many regions creates economic vulnerabilities and limits resilience in global feed supply chains [4].

In addition to sustainability concerns, conventional meat from monogastric animals often lacks desirable nutritional attributes. One major limitation is its low levels of omega-3 (n-3) polyunsaturated fatty acids (PUFAs) [5], which are essential for reducing inflammation, supporting cardiovascular health and enhancing brain function [6]. Traditional monogastric diets lack the precursors needed to elevate omega-3 PUFA levels in meat, resulting in a fatty acid profile dominated by omega-6 (n-6) PUFAs, which can contribute to pro-inflammatory states when consumed in excess [7]. Moreover, conventional meat has limited antioxidant capacity due to insufficient levels of compounds such as vitamin E, selenium and polyphenols. These deficiencies increase susceptibility to lipid oxidation, which compromises meat quality, reduces shelf life and diminishes its functional health benefits [2]. Additionally, high levels of saturated fats and cholesterol in conventional meat raise public health concerns due to their association with cardiovascular disease risk [8].

The integration of novel feed ingredients offers a relevant approach to improving meat quality and nutritional value while supporting sustainability. Insect meals, such as black soldier fly larvae, are rich in digestible protein and essential amino acids, enhancing growth performance and meat quality [9]. Microalgae, including *Chlorella vulgaris* and *Schizochytrium* spp., provide high levels of omega-3 fatty acids, antioxidants and carotenoids, improving the meat’s fatty acid profile, oxidative stability and shelf life [2]. Similarly, seaweeds like *Laminaria digitata* contribute essential minerals, iodine and bioactive compounds, further enhancing meat quality and stability [7]. Biofortified grains enriched with micronutrients such as zinc and selenium, as well as flaxseed and selenium-enriched wheat, address nutritional deficiencies, improving feed and meat quality [10]. Agro-industrial residues, including grape pomace and brewer’s spent grains, are cost-effective feed options rich in polyphenols and moderate protein, enhancing oxidative stability and sensory traits while supporting circular economy principles [11].

These novel feed ingredients address nutritional gaps and align with circular economy principles. For instance, by utilizing waste streams, such as grape pomace or olive oil by-products, they reduce environmental impacts while contributing to sustainable agricultural systems [12,13]. Additionally, biofortified crops offer a means of naturally increasing the micronutrient content of meat, potentially improving human diets and addressing global nutrient deficiencies [3].

Despite their potential, the adoption of novel feed ingredients faces significant challenges, including regulatory barriers, cost-effectiveness and variability in nutritional composition. Additionally, ensuring consistent palatability and digestibility for animals remains a key hurdle. Addressing these issues through standardized processing methods, supportive policies and cost-reduction strategies is essential for the successful integration of these ingredients into livestock diets [14].

This review examines the impact of novel feed ingredients on the quality and nutritional attributes of meat from monogastric animals. A systematic search in Google Scholar, PubMed, Scopus and Web of Science focused on terms like “novel feed ingredients”, “monogastric diets”, “meat quality” and ”meat nutritional value". Priority was given to recent studies on ingredients such as insect meal, microalgae, seaweeds, fermented by-products, biofortified grains and agro-industrial residues, excluding papers from non-indexed or non-English journals and those over 10 years old. This review analyses how these ingredients improve the meat’s nutritional profile, antioxidant content and sensory traits like flavour and texture. It also explores challenges and opportunities, including regulatory, economic and technical considerations, offering insights into sustainable livestock production and enhanced meat quality.

## 2. Overview of Novel Feed Ingredients

### 2.1. Definition and Categories

Novel feed ingredients for monogastric animals represent a transformative approach to improving meat quality, nutritional value and sustainability. These ingredients include insect meals, microalgae, seaweeds, fermented by-products, biofortified grains and agro-industrial residues. Each category provides unique bioactive properties, such as omega-3 fatty acids, antioxidants, essential amino acids and functional compounds, enhancing animal health and meat quality while reducing the environmental impact.

#### 2.1.1. Insect Meals

Insect meals, derived from species such as black soldier fly larvae (*Hermetia illucens*) and mealworms (*Tenebrio molitor*), are nutrient-dense feed ingredients providing 40–60% digestible protein and essential amino acids like lysine and methionine [14,15,16]. These meals also contain chitin, a bioactive fibre that promotes gut health and immunity, although enzymatic treatment may be required to enhance digestibility.

As a sustainable alternative to conventional protein sources, insect farming minimizes land and water use and utilizes organic waste, contributing to circular economy principles and reducing environmental footprints [17]. Studies show that replacing 10–20% of soybean meal with insect meal in poultry diets improves growth performance, feed efficiency and meat quality, without adverse health effects [15]. Additionally, insect meals (Tenebrio *molitor* larvae) are rich in functional lipids, such as lauric acid, and trace minerals like zinc, further supporting animal health and meat quality [9].

Insect meals offer a sustainable, high-quality protein source that aligns with global sustainability goals while enhancing the nutritional and sensory attributes of meat. In pigs, insect meal has shown promising results in improving growth performance and meat quality. For example, replacing 10–15% of soybean meal with black soldier fly larvae meal in pig diets was reported to enhance amino acid digestibility and improve pork’s sensory traits, including tenderness and juiciness [15]. Additionally, studies have highlighted its potential to improve gut health through its bioactive components like chitin, supporting overall animal performance and meat quality [14].

#### 2.1.2. Microalgae

Microalgae, such as *Chlorella vulgaris*, *Schizochytrium* spp. and Spirulina (*Limnospira platensis*), are notable for their high nutritional content and diverse applications in animal feed [2,17]. Spirulina is particularly rich in proteins, providing up to 60–70% protein content, along with a well-balanced amino acid profile. Additionally, Spirulina contains bioactive compounds such as phycocyanin and carotenoids, which exhibit antioxidant and anti-inflammatory properties, contributing to improved meat quality and stability [18]. In pigs, dietary supplementation with microalgae has been shown to enhance meat quality and nutritional value. For instance, the inclusion of Schizochytrium spp. at 2–3% in pig diets significantly improved the omega-3 fatty acid content in pork while reducing the omega-6/omega-3 ratio, aligning with human health recommendations [19]. Similarly, Spirulina supplementation increased antioxidant levels in pork, enhancing oxidative stability and shelf life [18]. These findings highlight the dual benefits of microalgae in improving both animal health and meat quality while contributing to sustainable farming practises.

In terms of lipid content, microalgae like *Schizochytrium* spp. are especially valued for their omega-3 fatty acids (docosahexaenoic acid, DHA; and eicosapentaenoic acid, EPA), which constitute 30–50% of their total lipids [19]. These long-chain fatty acids improve the omega-6/omega-3 ratio in meat, promoting cardiovascular and anti-inflammatory health benefits for consumers. Similarly, Spirulina contributes functional lipids and pigments like beta-carotene, enhancing meat colour and oxidative stability.

The dietary inclusion of 1–2% microalgae, including Spirulina, has been shown to significantly enrich the nutritional profile of broiler meat, increasing omega-3 content and antioxidant levels without compromising sensory attributes such as flavour or texture [18,19]. The combined benefits of Spirulina and other microalgae make them versatile and valuable feed additives for sustainable livestock production.

#### 2.1.3. Seaweeds

Seaweeds, such as *Laminaria digitata* and *Ulva* spp., are nutrient-rich feed ingredients that provide essential minerals (iodine, selenium and zinc), vitamins (A, D and E) and bioactive compounds like laminarin and fucoidan [19,20]. These compounds offer significant prebiotic and antioxidant benefits, contributing to improved gut health and meat quality. Including 3–5% seaweed in piglet diets has been shown to increase iodine content in pork by up to 45%, enhancing micronutrient density and addressing dietary deficiencies while extending meat shelf life [19]. The antioxidant properties of seaweed compounds also improve oxidative stability, helping to maintain meat flavour, tenderness and sensory appeal [20].

From a sustainability perspective, seaweed farming plays a critical role in nutrient recycling, carbon sequestration and mitigating eutrophication in marine ecosystems. These environmental benefits align with circular economy principles, making seaweeds an eco-friendly alternative to conventional feed ingredients. Furthermore, seaweed supplementation enriches meat fatty acid profiles, particularly through omega-3 incorporation, and can partially replace traditional feed components such as soybean meal. This highlights its potential as a cost-effective and sustainable dietary supplement for livestock production [21].

#### 2.1.4. Fermented By-Products

Fermentation processes transform agricultural residues into highly bioactive and digestible feed components, offering sustainable solutions for livestock nutrition. Examples include soybean hulls, grape pomace and citrus peels, whose anti-nutritional factors, such as phytic acid and tannins, are significantly reduced during fermentation. These processes enrich the feed with bioactive compounds like polyphenols, organic acids and probiotics, which enhance nutrient absorption, gut health and meat quality [10,22].

For instance, fermenting soybean hulls raises their protein content to 25–35%, which in turn enhances growth rates and amino acid absorption in monogastric diets. Grape pomace fermentation, when included at 5–7% in pig diets, enhances the oxidative stability and sensory attributes of meat, such as flavour and tenderness. Recent findings from Sun et al. [22] emphasize the broader application of fermented feeds, including reductions in oxidative stress, improved feed efficiency and their potential to substitute antibiotic growth promoters in monogastric production systems. These developments align with circular economy principles by reducing waste and converting by-products into valuable nutritional resources.

#### 2.1.5. Biofortified Grains

Biofortified grains are crops that have been genetically or agronomically enhanced to increase their nutrient content, aiming to address dietary deficiencies and improve livestock and human nutrition. They can enhance essential nutrients like alpha-linolenic acid (ALA), selenium, zinc and iron, benefiting livestock production and meat quality [23]. ALA, a precursor to omega-3 fatty acids like DHA and EPA, enriches the fatty acid profile of meat, creating a healthier balance. Including 10–15% flaxseed in monogastric diets has demonstrated improvements in omega-3 deposition and reductions in omega-6 levels, aligning with dietary health recommendations [5]. Selenium- and zinc-enriched cereals also enhance animal health and address global micronutrient deficiencies in meat consumers.

Dhaliwal et al. [23] highlight biofortification’s sustainability potential, particularly in combating hidden hunger in developing regions. Agronomic biofortification, such as applying micronutrient fertilizers and genetic improvements, has produced nutrient-dense staple crops. These grains enhance livestock mineral intake while reducing the reliance on external supplements, offering a cost-effective, scalable solution for improving meat quality and promoting sustainable livestock production [23].

#### 2.1.6. Agro-Industrial Residues

Agro-industrial residues, such as grape pomace, olive pomace, brewer’s spent grains and citrus peelings, offer sustainable and cost-effective feed options [11,24]. These by-products are rich in fibres, polyphenols, antioxidants and moderate protein levels, enhancing meat lipid stability, flavour and oxidative stability. Grape pomace, with its high polyphenol and dietary fibre content, improves oxidative stability and sensory traits like flavour and tenderness when included at 5–7% in pig diets, while also extending meat shelf life [24]. Similarly, the inclusion of up to 15% of brewer’s spent grains enhances the meat’s flavour and antioxidant properties without impairing growth performance [11].

Using these residues supports circular economy principles by reducing feed costs and valorising waste streams. For example, olive pomace enhances lipid stability in meat, while citrus peel residues, rich in flavonoids, promote gut health and improve meat quality traits in poultry. These versatile by-products align sustainability goals with enhanced livestock production efficiency [11,24].

### 2.2. Nutritional Composition

The nutritional composition of novel feed ingredients provides a significant advantage over conventional feed components, addressing dietary deficiencies in traditional livestock diets and improving meat quality.

#### 2.2.1. Proteins and Amino Acids

Insect meals, such as black soldier fly larvae (*Hermetia illucens*), are notable for their high protein content (40–60%) and a well-balanced amino acid profile, including lysine and methionine, which are essential for the growth and development of monogastric animals [14,16]. These attributes make insect meals a competitive alternative to traditional protein sources like soybeans. Moreover, studies show that replacing 15–20% of soybean meal with insect meal in broiler diets maintains or improves growth performance while enhancing lysine deposition in meat [15]. A unique advantage of insect meals is the presence of chitin, a bioactive fibre that supports gut health and immune function, although enzymatic treatments may be required to maximize its digestibility [14].

Microalgae, including *Chlorella vulgaris*, *Schizochytrium* spp. and Spirulina, are recognized for their high protein content, ranging from 50% to 70%. These proteins are rich in leucine, arginine and other essential amino acids critical for muscle development and recovery [17,18]. While microalgae naturally have rigid cell walls that limit digestibility, advancements in processing methods like enzymatic hydrolysis and pulsed electric field technology have significantly improved protein bioavailability, facilitating their integration into monogastric diets [25]. Among microalgae, Spirulina also offers unique bioactive compounds with additional health-promoting properties, further enhancing its utility as a high-value feed ingredient.

Fermented by-products, such as soybean hulls treated with *Bacillus subtilis*, offer another sustainable protein option by addressing anti-nutritional factors like phytic acid. These by-products yield 25–35% protein and improve amino acid absorption, particularly in pigs. For example, diets including up to 20% fermented soybean hulls significantly enhanced feed efficiency, growth rates and meat quality [10,22]. Fermentation further enriches these feeds with organic acids and enzymes, improving nutrient bioavailability and gut health, making them a versatile and cost-effective choice.

When comparing these protein sources, insect meals stand out for their sustainability and nutrient density, leveraging organic waste while providing a complete amino acid profile. Microalgae, particularly Spirulina and *Chlorella vulgaris*, are unmatched in protein concentration and bioactive diversity, though their current cost and processing challenges limit their widespread adoption. Fermented by-products excel in cost-effectiveness and dual functionality, addressing anti-nutritional factors and improving gut health. Together, these novel ingredients provide complementary benefits, enabling tailored feed solutions to meet specific production goals.

#### 2.2.2. Omega-3 Fatty Acids and Lipid Profile

The incorporation of omega-3 fatty acids into livestock diets is a relevant advancement in enhancing the nutritional value of meat [26]. Microalgae, particularly *Schizochytrium* spp. and *Chlorella vulgaris*, are exceptional sources of omega-3 fatty acids, including DHA and EPA. These long-chain omega-3 PUFAs improve the omega-6/omega-3 ratio in meat, reducing inflammation and supporting cardiovascular health in humans. Microalgae contain lipid levels of 20–40%, with 30–50% of these lipids comprising omega-3 fatty acids [17]. Mendes et al. [27] demonstrated that cumulative intakes of 20 g of *Chlorella vulgaris* per bird enriched meat with omega-3 fatty acids and carotenoids while improving oxidative stability and flavour.

Seaweeds, such as *Laminaria digitata*, contribute omega-3 fatty acids and functional lipids, though in smaller quantities compared to microalgae. Additionally, seaweeds contain bioactive compounds like fucoxanthin, which influence lipid metabolism and enhance fatty acid profiles in meat [19]. According to Costa et al. [17], including 3–5% *Laminaria* in monogastric diets improved omega-3 levels and reduced lipid peroxidation, contributing to better meat stability.

Biofortified grains, such as flaxseed-enriched cereals, provide ALA, a precursor for DHA and EPA synthesis in monogastric animals. Including 10–15% flaxseed in pig diets significantly increases omega-3 deposition in pork while reducing omega-6 PUFAs, aligning with dietary recommendations for a balanced fatty acid profile [23]. Tang et al. [5] reported that biofortified grains not only improved the nutritional composition of meat but also reduced pro-inflammatory markers.

While microalgae excel in DHA and EPA enrichment, their high cost restricts their widespread use. Seaweeds offer a balanced middle ground, enhancing omega-3 content with added antioxidant benefits and environmental advantages, albeit with less lipid concentration than microalgae. Biofortified grains, offering ALA, are a cost-effective alternative but rely on the animal’s metabolic conversion efficiency, which is often suboptimal. This comparative understanding underscores the importance of choosing feed ingredients based on specific production goals, such as optimizing fatty acid profiles, balancing costs or achieving sustainability targets.

#### 2.2.3. Antioxidants and Stability

Antioxidants are essential for extending meat shelf life and preserving quality by mitigating lipid oxidation, a primary cause of spoilage and flavour deterioration. Novel feed ingredients offer unique antioxidant properties that enhance meat stability, with each source contributing distinct bioactive compounds and mechanisms.

Microalgae, such as *Chlorella vulgaris*, are rich in carotenoids, including astaxanthin and lutein, which are potent antioxidants [17]. These compounds significantly reduce lipid oxidation and improve oxidative stability. Mendes et al. [27] demonstrated that incorporating *Chlorella vulgaris* into poultry diets reduced lipid peroxidation by up to 40%, leading to extended shelf life and improved meat quality. Additionally, the antioxidative capacity of microalgae complements its nutritional benefits, making it a versatile feed ingredient.

Seaweeds, including species such as *Laminaria digitata* and *Ulva* spp., contribute to antioxidative effects through bioactives like laminarin and fucoidan. These compounds not only reduce oxidative stress but also act as prebiotics, enhancing gut health in livestock [19]. Ribeiro et al. [7] found that incorporating 3–5% seaweed into pig diets improved oxidative stability and prolonged meat storage life, emphasizing its dual role in nutritional and functional enhancement.

Agro-industrial residues, such as grape pomace and olive cake, provide a diverse array of antioxidants, particularly polyphenols, which play a crucial role in reducing oxidative stress and enhancing meat quality [24]. Vastolo et al. [11] highlighted that grape pomace, included at 5–7% in monogastric diets, effectively extends meat shelf life and improves sensory traits such as flavour and tenderness. The fibre content in residues like olive cake also modulates gut microbiota, indirectly supporting nutrient absorption and animal health, further contributing to meat stability.

Among these novel feed ingredients, microalgae stand out for their high concentrations of carotenoids, making them particularly effective in reducing lipid peroxidation and enhancing meat stability. Agro-industrial residues, while offering lower antioxidant concentrations compared to microalgae, provide additional benefits through their polyphenols and fibre content, which support both oxidative stability and gut health. Seaweeds, with its unique bioactive compounds and prebiotic properties, strike a balance by contributing to oxidative stability and to the overall well-being of livestock. The choice of antioxidant-rich feed ingredients should align with specific production goals, whether prioritizing meat shelf life, cost-effectiveness or added nutritional benefits.

#### 2.2.4. Minerals and Vitamins

The inclusion of novel feed ingredients provides an effective means of enriching the mineral and vitamin content of meat, addressing significant micronutrient deficiencies in human diets while enhancing the meat’s quality and nutritional value.

Microalgae, such as *Chlorella vulgaris*, are a potent source of vitamins and minerals. These organisms contain high levels of vitamins E and C, which act as natural antioxidants, improving meat oxidative stability and extending shelf life. Costa et al. [17] emphasized that these vitamins enhance the meat’s functional and sensory properties, such as texture and colour, increasing its appeal to health-conscious consumers. The high bioavailability of these micronutrients in microalgae makes it a valuable addition to monogastric diets.

Seaweeds, such as *Laminaria digitata*, are very rich in essential minerals, including iodine, selenium and zinc, as well as vitamins A, D and E. These nutrients are vital for improving both livestock health and the nutritional composition of meat. Incorporating 3–5% *Laminaria digitata* in monogastric diets has been shown to increase iodine levels in pork by up to 45%, effectively addressing iodine deficiencies in human diets through moderate meat consumption [7]. Additionally, the dual functionality of seaweed in reducing oxidative stress and enhancing sensory qualities, such as flavour and tenderness, makes it a sustainable and nutritionally impactful feed component.

Biofortified grains, such as zinc-enriched wheat and iron-fortified rice, offer an innovative strategy for enhancing the micronutrient profiles of livestock feed. These grains improve the nutritional composition of meat by boosting its zinc, selenium and iron content. Dhaliwal et al. [23] reported that biofortification significantly increases these essential micronutrients, making biofortified grains an effective and scalable option for addressing consumer health concerns. Incorporating biofortified grains in feed aligns with global public health goals by reducing micronutrient deficiencies through sustainable livestock production.

Among these novel feed ingredients, seaweed excels in providing a diverse range of essential minerals and fat-soluble vitamins, offering comprehensive nutritional benefits. Microalgae stand out for their potent antioxidant properties due to high vitamin E and C concentrations, which directly enhance meat stability and shelf life. In contrast, biofortified grains focus primarily on specific micronutrient deficiencies, such as zinc and iron, making them ideal for targeted dietary interventions. While seaweed and microalgae offer additional functional benefits, such as oxidative stability and sensory enhancement, biofortified grains present a cost-effective solution with broad applicability. Selecting the optimal ingredient depends on the specific production goals, such as addressing consumer health priorities or improving meat quality and longevity.

#### 2.2.5. Combined Nutritional Benefits

The integration of novel feed ingredients delivers synergistic improvements to meat quality, nutritional value and livestock performance, addressing the key limitations of traditional diets while promoting sustainability.

Novel feeds enrich meat with high-quality proteins, omega-3 fatty acids, antioxidants and essential micronutrients. Insect meals provide digestible proteins with balanced amino acid profiles, while microalgae and seaweed deliver functional lipids, such as DHA and EPA, which improve the omega-6/omega-3 ratio and enhance human cardiovascular health. Fermented by-products contribute bioactive compounds that reduce oxidative stress, and biofortified grains supply key micronutrients like selenium and zinc to address common dietary deficiencies.

These ingredients enhance feed digestibility and nutrient absorption, improving growth rates and overall animal health. Functional components, such as chitin in insect meals and prebiotic compounds in seaweed, support gut health and immunity, leading to better animal performance and meat quality.

By incorporating sustainable and nutrient-rich alternatives, such as agro-industrial residues, insect meals and biofortified grains, these feeds reduce environmental impacts, including land and water use and align with circular economy principles. This holistic approach enhances meat quality and supports the demand for environmentally responsible production systems.

These novel feed ingredients redefine livestock nutrition by combining superior nutritional enhancements with sustainability, offering a comprehensive solution for evolving consumer and environmental needs. Table 1 highlights the dietary contributions of each ingredient, showcasing their complementary roles in improving monogastric diets and meat quality.

## 3. Impacts on Meat Quality and Nutritional Value

### 3.1. Sensory Characteristics

Novel feed ingredients significantly enhance sensory attributes such as flavour, tenderness, texture and colour, which are pivotal for consumer satisfaction and marketability. Ingredients rich in polyphenols, like grape by-products, improve the oxidative stability and sensory profiles of meat. For instance, incorporating 5–7% grape pomace into pig diets reduces lipid oxidation and enhances flavour and tenderness, resulting in extended shelf life and consumer appeal [24]. These findings demonstrate the dual role of polyphenols in augmenting both the sensory and functional properties of meat.

Microalgae contribute unique pigments, such as carotenoids, which enhance meat colour, a critical aspect of consumer preference. For example, Ribeiro et al. [19] observed that the dietary inclusion of 2% microalgae significantly improved the yellow and red pigmentation of broiler meat while maintaining tenderness and juiciness. This enhancement boosts visual appeal without compromising texture. Additionally, bioactive compounds in microalgae, like astaxanthin, enhance water-holding capacity and oxidative stability, further supporting meat quality.

Seaweed offers complementary benefits through its bioactive compounds, such as laminarin and fucoidan, which reduce spoilage and bolster water retention. Supplementing monogastric diets with 3–5% seaweed has been shown to improve meat stability and extend storage life [7]. These prebiotic effects also enhance gut health in livestock, indirectly supporting overall sensory improvements in meat products.

When comparing the sensory contributions of novel feed ingredients, microalgae excel in improving meat colour and oxidative stability due to their carotenoid content. Grape pomace is unparalleled in flavour enhancement and tenderness due to its high polyphenol concentration. Seaweeds, with their water-retention and spoilage-reduction capabilities, are particularly effective in extending shelf life and maintaining sensory appeal during storage. Collectively, these ingredients offer a versatile toolkit for addressing diverse sensory demands, aligning with both consumer preferences and production goals.

### 3.2. Nutritional Improvements

#### 3.2.1. Protein Quality

Novel feed ingredients such as insect meals and fermented by-products significantly enhance the protein content and amino acid profiles in meat. Insect meals, particularly those derived from black soldier fly larvae (*Hermetia illucens*), are rich in digestible protein (40–60%) and essential amino acids like lysine and methionine. Hong et al. [15] demonstrated that replacing 10–20% of soybean meal with insect meal in broiler diets improved protein digestibility and lysine content, highlighting insect meal as a sustainable and effective protein source without compromising growth performance or meat quality.

Fermented by-products, such as soybean hulls treated with *Bacillus subtilis*, offer improved protein availability by reducing anti-nutritional factors such as phytic acid. These by-products contain 25–35% protein and enhance amino acid absorption and meat protein content. Wongputtisin et al. [10] reported that including up to 20% fermented soybean hulls in pig diets enhanced feed efficiency and growth rates. Sun et al. [22] further emphasized that fermentation introduces bioactive compounds like organic acids and enzymes, which improve gut health, nutrient absorption and overall meat quality. This dual role of increasing protein availability and providing functional bioactives underscores the transformative potential of fermented by-products in monogastric diets.

#### 3.2.2. Fatty Acid Enrichment

Incorporating omega-3-rich feed ingredients such as microalgae, seaweed and biofortified grains enhances the fatty acid profile of meat, improving its nutritional value. Seaweed, such as *Laminaria digitata*, is rich in omega-3 fatty acids and iodine, making it a valuable dietary addition for enhancing meat quality. Ribeiro et al. [7] found that feeding pigs with a diet containing 5% seaweed increased omega-3 fatty acid levels in pork while reducing the omega-6/omega-3 ratio, aligning with human dietary recommendations.

Microalgae, such as *Schizochytrium* spp., are exceptional sources of DHA and EPA, long-chain omega-3 fatty acids critical for cardiovascular and anti-inflammatory health. Ribeiro et al. [19] demonstrated that including 2% microalgae in broiler diets increased DHA content in chicken breast meat by over 70%, without negatively affecting growth performance or sensory traits. This highlights the efficiency of microalgae as a targeted feed ingredient for omega-3 enrichment.

Biofortified grains, particularly flaxseed-enriched cereals, are high in ALA, a precursor to DHA and EPA. Tang et al. [5] found that including 15% flaxseed in pig diets significantly increased omega-3 PUFA content in pork while reducing omega-6 levels, improving the meat’s anti-inflammatory properties and aligning with dietary health goals.

#### 3.2.3. Antioxidants and Oxidative Stability

Antioxidants play a pivotal role in enhancing meat stability and extending shelf life by reducing lipid oxidation. Microalgae, rich in carotenoids such as astaxanthin and lutein, provide strong antioxidative effects. Mendes et al. [27] reported that including *Chlorella vulgaris* in poultry diets reduced lipid peroxidation by 40%, significantly improving oxidative stability and sensory traits.

Seaweeds also contribute antioxidative compounds, including polyphenols, fucoxanthin and laminarin, which protect against oxidative stress. Ribeiro et al. [7] described that incorporating 3–5% *Laminaria digitata* into pig diets reduced lipid oxidation and enhanced meat stability during storage.

Agro-industrial residues, such as grape pomace, are another potent antioxidant source, offering high levels of polyphenols that stabilize lipids and improve meat flavour. Alfaia et al. [24] reported that feeding grape pomace to pigs improved oxidative stability by 30%, contributing to longer shelf life and superior sensory attributes.

This antioxidant synergy across novel feed ingredients highlights their importance in improving the functional and sensory qualities of meat while reducing spoilage.

#### 3.2.4. Micronutrient Fortification

Novel feed ingredients such as seaweed and microalgae also enhance meat micronutrient content. Seaweed is an excellent source of iodine, selenium and zinc, along with vitamins A, D and E. Ribeiro et al. [7] reported that supplementing pig diets with 3–5% seaweed increased the iodine content in pork by 45%, effectively addressing iodine deficiencies in human diets through modest meat portions.

Microalgae contribute significantly to vitamin fortification, particularly vitamins E and C, which act as antioxidants to enhance meat stability and shelf life. Ribeiro et al. [19] reported that broiler diets supplemented with 2% microalgae increased vitamin E content in chicken meat, boosting its nutritional and health benefits for consumers.

#### 3.2.5. Comparative Benefits

Insect meals excel in providing high-quality protein (40–60%) and essential amino acids like lysine and methionine, supporting growth and muscle development in monogastric animals while promoting sustainability. Fermented by-products enhance protein bioavailability and introduce functional bioactives like polyphenols and organic acids, improving nutrient absorption, gut health and antioxidant capacity.

Microalgae are unmatched in omega-3 enrichment, particularly DHA and EPA, which benefit cardiovascular health. Their carotenoids, such as astaxanthin, act as potent antioxidants, enhancing meat stability and shelf life. Seaweeds complement these benefits by offering moderate omega-3 levels alongside essential minerals (iodine, selenium and zinc) and antioxidant bioactives like fucoxanthin, improving meat quality and sensory traits.

Biofortified grains enhance the fatty acid profile through ALA and address micronutrient deficiencies like zinc and selenium. Together, these ingredients provide complementary advantages, improving protein quality, fatty acid balance, micronutrient levels, and antioxidant content, benefiting both livestock performance and consumer health while aligning with sustainability goals.

Table 2 summarizes the major impacts of novel feed ingredients on key meat quality and nutritional attributes.

## 4. Practical Considerations

### 4.1. Animal Acceptance and Digestibility

The successful integration of novel feed ingredients into monogastric diets requires the addressing of challenges in palatability and digestibility, which is critical for their effectiveness.

Palatability is a primary hurdle when introducing unconventional feed ingredients like insect meals or microalgae. Animals may initially reject feeds due to strong odours or unfamiliar textures. For instance, black soldier fly larvae meal exhibits an odour profile that can be mitigated through pre-treatment methods such as defatting or flavour masking. Studies, such as those conducted by Mulia et al. [28], demonstrate that incorporating defatted BSF larvae meal into pig diets does not negatively affect feed intake, indicating that proper pre-treatment can enhance acceptance.

Microalgae, despite their nutrient density, can face similar palatability issues due to their high lipid content and unique taste profiles. Advances in processing, such as encapsulation and enzymatic treatments [17], have been effective in overcoming these barriers, ensuring consistent feed intake without adverse effects on animal performance.

The digestibility of novel feed ingredients determines their nutritional contribution. Components like chitin in insect meals can reduce nutrient absorption due to its structural complexity. However, processing techniques such as enzymatic hydrolysis and fermentation significantly enhance nutrient release. Sajid et al. [16] reported a 20–25% improvement in protein digestibility and amino acid availability in poultry-fed proteolytic enzyme-treated insect meal.

Fermented agro-industrial by-products, such as soybean hulls treated with *Bacillus subtilis*, offer enhanced digestibility by reducing anti-nutritional factors like phytic acid. Wongputtisin et al. [10] highlighted that including up to 20% fermented soybean hulls in pig diets improved amino acid absorption, providing a cost-effective protein source with superior digestibility. Furthermore, microbial fermentation enriches feeds with organic acids and enzymes, enhancing gut health and nutrient absorption.

Among novel feed ingredients, insect meals excel in sustainability and protein digestibility, particularly when treated to reduce chitin content. Fermented by-products provide the dual benefit of enhanced bioavailability and gut health, while microalgae stand out for their lipid and antioxidant profiles, albeit with higher processing requirements to overcome palatability challenges. Together, these ingredients offer complementary advantages, enabling tailored solutions for monogastric nutrition.

### 4.2. Regulatory and Safety Issues

The regulatory framework for novel feed ingredients varies significantly across regions, reflecting differences in safety standards, evaluation protocols and market readiness. In the European Union (EU), the Novel Food Regulation (EU) 2015/2283 provides a comprehensive framework for the approval of ingredients such as insect meal and algae-based feeds. This regulation mandates a thorough safety evaluation by the European Food Safety Authority (EFSA), addressing concerns such as allergenicity, microbiological contamination, heavy metal residues and the transfer of feedborne toxins to meat products. For instance, insect-derived feeds like *Tenebrio molitor* meal are subject to allergenicity assessments due to proteins that may trigger hypersensitivity reactions in humans [29].

In the United States, the Food and Drug Administration (FDA) governs novel feed ingredients through its Generally Recognized as Safe (GRAS) certification process. This approach evaluates the safety of feed additives based on scientific evidence but often lacks the comprehensive guidelines seen in the EU. The absence of harmonized global regulations poses challenges for manufacturers seeking international market access, complicating the approval process and increasing compliance costs.

A critical safety concern is the substrate used for rearing insects or cultivating algae, which can introduce contaminants such as pathogens, heavy metals or pesticide residues [28]. Klerx et al. [30] emphasized the necessity of rigorous quality control systems to monitor organic waste streams used for insect rearing, minimizing the risk of contamination. Similarly, algae cultivation in open systems may expose products to environmental pollutants, requiring advanced screening and decontamination methods. The development of standardized protocols for substrate monitoring and ingredient processing is essential to mitigate these risks and ensure consumer safety.

Global collaboration to harmonize regulatory standards, enhance transparency and establish traceability systems could reduce barriers to the adoption of novel feed ingredients. These measures would promote safety and support the scalability and international trade of sustainable livestock feed solutions.

### 4.3. Economic Feasibility

The economic viability of integrating novel feed ingredients into monogastric diets is a critical factor for their adoption. Ingredients such as insect meals, microalgae, seaweeds, fermented by-products and agro-industrial residues present varying cost profiles and opportunities for cost reduction while contributing to sustainability goals.

#### 4.3.1. Insect Meals

Insect meal, particularly black soldier fly larvae, is a sustainable and nutritionally dense alternative to traditional protein sources like soybean meal and fishmeal. However, its production costs, ranging from $2.00 to $3.00 per kg, remain a barrier compared to soybean meal ($0.40–$0.50/kg) and fishmeal ($1.50–$2.00/kg). Up to 30% of these costs are attributed to processing steps like drying and grinding. Adopting automated rearing systems and utilizing agro-industrial waste as rearing substrates could reduce costs by 40–60% [14]. Successful examples include Kenyan farmers reducing feed costs by 15–25% by replacing 50–100% of fishmeal with BSF larvae meal [28].

#### 4.3.2. Microalgae

Microalgae, such as *Chlorella vulgaris* and *Schizochytrium* spp., are prized for their omega-3 fatty acids and other nutritional benefits but remain cost-intensive. Current production costs range from $5.00 to $8.00 per kg due to energy-intensive cultivation and drying methods. However, integrating microalgae production with wastewater treatment systems has shown promise for reducing costs by 30–50% while maintaining the nutritional quality. These systems offer the dual benefit of environmental remediation and high-value feed production. In aquaculture, microalgae have replaced up to 50% of fish oil in diets, underscoring their economic feasibility in high-value applications despite current cost challenges [19].

#### 4.3.3. Seaweeds

Seaweeds, particularly *Laminaria digitata* and *Ulva* spp., provide nutritional and environmental benefits. Naturally harvested seaweed costs $0.50–$2.00 per kg, with intensive cultivation potentially raising these costs. However, the dual role of seaweed in nutrient enrichment and environmental remediation, such as reducing eutrophication and sequestering carbon, offsets its higher production expenses. For example, supplementing pig diets with 3–5% *Laminaria* has been shown to improve iodine content in pork and enhance omega-3 fatty acid levels, aligning with both economic and sustainability goals [7].

#### 4.3.4. Fermented By-Products

Fermented by-products have emerged as cost-effective solutions in monogastric nutrition. Sun et al. [22] highlighted that locally available agricultural residues, such as soybean hulls and citrus peels, can be transformed into high-value feed ingredients through fermentation, significantly reducing feed costs. These by-products enhance nutrient bioavailability and introduce bioactive compounds, such as organic acids, which improve feed efficiency and animal performance. This strategy is particularly valuable in regions with limited access to imported feed components, offering an economical and sustainable alternative.

#### 4.3.5. Agro-Industrial By-Products

Agro-industrial residues are among the most economical novel feed ingredients due to their low cost and wide availability. Vastolo et al. [11] emphasized that these by-products, such as grape pomace and brewer’s spent grains, align with circular economy principles by repurposing waste into valuable feed components. Grape pomace, priced at $0.10–$0.20 per kilogram, not only reduces feed costs but also improves oxidative stability and sensory attributes when included at 5–7% in pig diets [24]. Brewer’s spent grains, available in significant quantities from the brewing industry, are another cost-effective option, particularly in regions with established beverage production.

#### 4.3.6. Market Acceptance

Market acceptance remains pivotal for the economic viability of these ingredients. Surveys in Europe reveal that 67% of farmers are open to adopting insect- or microalgae-based feeds, provided that they are cost-competitive [31,32]. The rising consumer demand for organic and sustainable meat products further supports the potential for premium pricing. By scaling up production, integrating waste streams and optimizing cultivation methods, these novel feed ingredients are likely to achieve greater market penetration and economic feasibility within the next decade [33].

This diversified economic outlook underscores the importance of leveraging regional resources, advancing production technologies and aligning with sustainability principles to integrate novel feed ingredients effectively into monogastric diets.

## 5. Challenges and Opportunities

### 5.1. Technical Barriers

The adoption of novel feed ingredients into monogastric diets presents several technical challenges, particularly concerning nutrient bioavailability, processing costs and consistency in nutritional quality.

#### 5.1.1. Nutrient Bioavailability

Seaweed’s tough cell walls, rich in polysaccharides like alginates and cellulose, hinder nutrient release during digestion. Advanced processing techniques such as enzymatic hydrolysis, fermentation and thermal treatments have been developed to address this issue [20]. Enzymatic hydrolysis can enhance the bioavailability of iodine and omega-3 fatty acids by up to 40% [3]. Similarly, microalgae like *Chlorella vulgaris* possess rigid cell walls requiring disruption techniques, such as ultrasonic processing, which has been shown to improve nutrient release by 30% compared to untreated samples [18]. These advancements improve the nutritional efficacy of these ingredients, aligning them with the dietary needs of monogastric production systems.

#### 5.1.2. Processing Costs

High processing costs, particularly for energy-intensive ingredients like microalgae and seaweeds, remain a significant barrier to their widespread adoption. Traditional drying and harvesting methods contribute substantially to these expenses. However, cost-reduction innovations such as solar drying and integrated bioreactor systems have achieved energy savings of 25–35%, improving economic feasibility for large-scale applications [34]. Moreover, co-cultivating microalgae with wastewater treatment systems has demonstrated the potential to lower nutrient input costs while maintaining high yields, further supporting the sustainability and scalability of these systems.

#### 5.1.3. Consistency in Nutritional Quality

Achieving consistent nutritional profiles in novel feed ingredients is another challenge, as nutrient composition can vary based on cultivation conditions, substrate quality and processing methods. For instance, fluctuations in the quality of organic waste streams used for insect rearing can lead to variability in protein and fat content. Standardized production protocols and rigorous quality control measures are essential to mitigate these inconsistencies. Real-time monitoring technologies, highlighted by Huis et al. [35], offer promising solutions by ensuring uniform nutrient profiles across production batches, enhancing the reliability of these ingredients in livestock nutrition.

### 5.2. Sustainability Goals

The integration of novel feed ingredients into monogastric diets is a pivotal step toward reducing the environmental footprint of meat production. By utilizing sustainable resources, these ingredients address critical challenges in agriculture while promoting global sustainability objectives.

#### 5.2.1. Environmental Impact Reduction

Insect meals, such as black soldier fly larvae meal, are highly resource-efficient, requiring significantly less land, water and energy compared to traditional protein sources like soybean and fishmeal. Replacing 50% of conventional protein sources with insect meal has been shown to reduce greenhouse gas emissions by up to 30% [3]. Furthermore, insect farming utilizes agro-industrial waste streams, reducing overall waste while contributing to circular economy principles [14].

#### 5.2.2. Microalgae Cultivation

Microalgae, such as *Chlorella vulgaris* and *Schizochytrium* spp., are particularly noteworthy for their ability to enhance the nutritional quality of meat while supporting sustainability goals. Their cultivation requires minimal arable land and can be integrated with wastewater treatment systems, reducing nutrient inputs and recycling carbon. Ribeiro et al. [19] demonstrated that microalgae cultivation reduced resource use while providing high-value omega-3 fatty acids, aligning with eco-friendly livestock production practises.

#### 5.2.3. Seaweed Cultivation

Seaweed farming offers the dual benefit of nutrient recycling and carbon sequestration. By absorbing nitrogen and phosphorus from aquatic environments, seaweed mitigates eutrophication while providing a nutrient-dense feed component. McCauley et al. [36] reported that seaweed incorporation into monogastric diets reduced feed-related environmental impacts by 20–25%, largely due to its minimal resource demands and high nutritional density. Additionally, seaweed aligns with sustainable marine practises and can serve as a scalable solution to environmental challenges in livestock production.

#### 5.2.4. Agro-Industrial Residues

The use of agro-industrial residues, including grape pomace and brewer’s spent grains, is an exemplary model of waste valorisation. These by-products transform food industry waste into valuable livestock feed, reducing the reliance on conventional feed components. Vastolo et al. [11] highlighted that incorporating agro-industrial residues in monogastric diets not only lowers feed costs but also enhances the meat’s oxidative stability and flavour. The same authors [11] further emphasized the role of these residues in reducing greenhouse gas emissions and advancing circular economy principles within agricultural systems.

By incorporating sustainable feed ingredients like insect meals, seaweed, microalgae and agro-industrial residues, livestock systems can significantly reduce their environmental impact. These novel feed options optimize resource use, support waste valorisation and align with global sustainability goals, paving the way for eco-friendly livestock production.

### 5.3. Future Research Directions

Unlocking the full potential of novel feed ingredients requires a multi-faceted research approach involving genetic improvements, cost-effective processing, biofortification and comprehensive studies on quality and market acceptance.

#### 5.3.1. Genetic Improvements in Animals

Advancements in genetic and breeding technologies could improve the nutrient utilization efficiency in animals. For example, enhancing the omega-3 desaturase enzyme expression in pigs may increase long-chain omega-3 fatty acid content in pork, enriching its nutritional value [37]. Techniques like CRISPR/Cas9 could further enhance feed conversion and nutrient absorption, ultimately lowering feed costs and improving sustainability [38].

#### 5.3.2. Cost-Effective Processing and Biofortification

Adopting cost-effective technologies, such as fermentation and enzymatic treatments, is essential for scaling novel feeds. Fermented insect meal, for instance, has demonstrated improved digestibility and reduced anti-nutritional factors, offering a sustainable soybean meal alternative [14]. Integrating agro-industrial waste for insect rearing also supports circular economy principles, cutting costs and enhancing sustainability.

The biofortification of grains with omega-3 fatty acids and essential micronutrients like selenium could improve meat quality and consumer health. Expanding these efforts to diverse crops and climates, as well as integrating genetic and agronomic techniques, can improve livestock nutrition and meat quality [23].

#### 5.3.3. Long-Term Studies on Meat Quality and Consumer Acceptance

Long-term studies on the impacts of novel feeds are crucial for assessing meat quality, nutritional value and market acceptance. Research should address consumer perceptions of meat produced with alternative feeds, focusing on sensory and health attributes to encourage broader adoption [14,39].

#### 5.3.4. Case Studies of Successful Integration

Successful case studies offer valuable lessons on the integration of novel feed ingredients. For instance, using black soldier fly larvae in pilot projects has reduced feed costs by 20% without affecting performance or meat quality [14]. Similarly, European seaweed farming shows dual benefits, environmental remediation and high-quality feed production, which can serve as a replicable model elsewhere [7].

## 6. Conclusions

This review underscores the transformative potential of novel feed ingredients, mainly insect meals, microalgae, fermented by-products, biofortified grains and agro-industrial residues, in improving monogastric animal nutrition. These innovative ingredients significantly enhance meat quality by enriching omega-3 fatty acids and antioxidants and improving flavour, tenderness and colour, aligning with consumer demand for healthier foods. From a sustainability perspective, insect meals and agro-industrial by-products contribute to circular economy principles, while microalgae and seaweed aid in nutrient recycling and carbon sequestration, reducing the environmental impact of meat production. Biofortified grains also hold promise for improving the micronutrient profile of meat, helping address public health needs.

Challenges remain, particularly in reducing production costs, improving nutrient bioavailability and overcoming regulatory barriers. The high costs of insect meal and microalgae production are significant hurdles, but advancements in enzymatic treatments, fermentation and scalable production systems offer pathways for cost reduction and broader adoption. Moving forward, collaborative efforts among researchers, policymakers and industry are crucial. Key research areas should include optimizing feed formulations, reducing processing costs, improving consumer acceptance through sensory trials and conducting long-term studies on animal health and meat quality. Genetic and breeding strategies for enhancing nutrient uptake and feed efficiency also present promising opportunities for further innovation.

## Figures and Tables

**Table 1:** Comparison of specific nutritional benefits of novel feed ingredients.

Nutritional Aspect	Insect Meal [14,15,16]	Microalgae [18,19,27]	Seaweeds [7,20,21]	Fermented By-Products [10,22]	Biofortified Grains [5,23]	Agro-Industrial Residues [11,24]
Protein Quality	High-quality, digestible protein (40–60%), rich in lysine and methionine	High protein content (40–70%) with a complete amino acid profile, including arginine and leucine	Moderate protein (10–20%), varies by species	Improved digestibility *post*-fermentation (25–35%)	Moderate protein (15–25%), enhanced by biofortification	Moderate protein (15–20%), rich in fibre
Omega-3 Fatty Acids	Limited, primarily omega-6 with minor omega-3 content	High in DHA, EPA and functional lipids (up to 50% of total lipids); Spirulina contains GLA	Modest omega-3 levels, primarily contributes functional lipid	Limited unless enriched by substrate	Rich in ALA, a precursor for DHA/EPA synthesis	Limited unless associated with specific residues (e.g., grape seeds)
Antioxidants	Chitin provides mild oxidative protection	Rich in carotenoids (e.g., lutein and astaxanthin) and phycocyanin (Spirulina), reducing lipid peroxidation by 30–40%	High levels of polyphenols, vitamins A, E and bioactive compounds (e.g., fucoxanthin)	Polyphenols present, enhancing oxidative stability	Limited unless fortified	Rich in polyphenols, enhancing oxidative stability (e.g., grape pomace)
Mineral Content	Iron, zinc and phosphorus	Calcium, magnesium, iron and trace minerals; Spirulina is particularly rich in iron and potassium	Rich in iodine, selenium and zinc, enhancing mineral density in meat	Varies based on source; fermentation may enhance bioavailability	Selenium and zinc (fortification-dependent)	Limited to trace minerals depending on the residue type
Bioactive Compounds	Antimicrobial peptides and chitin boost immune function	Functional lipids and pigments, like astaxanthin and phycocyanin (Spirulina), improve oxidative stability	Laminarin and fucoidan with prebiotic and antioxidant effects	Probiotics and secondary metabolites (e.g., flavonoids)	None inherently, relies on external fortification	Polyphenols and fibres with prebiotic potential (e.g., brewer’s spent grains)

**Table 2:** Summary of major impacts of novel feed ingredients on meat quality and nutritional value.

Aspect	Ingredients	Impacts	Main References
Sensory Characteristics	Grape by-products, microalgae and seaweeds	Improved flavour, tenderness, colour, texture and oxidative stability	[7,19,24]
Functional Properties	Grape by-products, microalgae and seaweeds	Extended shelf life, reduced lipid peroxidation and enhanced water-holding capacity	[7,19,24]
Improved Protein Quality	Insect meal and fermented by-products	High protein digestibility (40–60%), better amino acid profiles and reduced anti-nutritional factors	[10,14,15,16,22]
Fatty Acid Enrichment	Microalgae, seaweeds and biofortified grains	Enriched omega-3 (DHA/EPA) and reduced omega-6/omega-3 ratio	[5,7,19]
Antioxidant Enhancement	Microalgae, seaweeds and agro-industrial residues	Reduction in lipid oxidation (up to 40%), higher oxidative stability and extended storage durability	[7,11,24,27]
Micronutrient Fortification	Seaweeds and microalgae	Higher iodine, selenium, zinc and vitamins A, D and E	[7,17,23]

## Data Availability

No new data were created or analyzed in this study. Data sharing is not applicable to this article.
